# Poverty and maternal mortality in Nigeria: towards a more viable ethics of modern medical practice

**DOI:** 10.1186/1475-9276-7-11

**Published:** 2008-04-30

**Authors:** Bolatito A Lanre-Abass

**Affiliations:** 1 Department of Philosophy, University of Ibadan, Ibadan, Nigeria

## Abstract

Poverty is often identified as a major barrier to human development. It is also a powerful brake on accelerated progress toward the Millennium Development Goals. Poverty is also a major cause of maternal mortality, as it prevents many women from getting proper and adequate medical attention due to their inability to afford good antenatal care. This Paper thus examines poverty as a threat to human existence, particularly women's health. It highlights the causes of maternal deaths in Nigeria by questioning the practice of medicine in this country, which falls short of the ethical principle of showing care.

Since high levels of poverty limit access to quality health care and consequently human development, this paper suggests ways of reducing maternal mortality in Nigeria. It emphasizes the importance of *care ethics*, an ethical orientation that seeks to rectify the deficiencies of medical practice in Nigeria, notably the problem of poor reproductive health services.

Care ethics as an ethical orientation, attends to the important aspects of our shared lives. It portrays the moral agent (in this context the physician) as a self who is embedded in webs of relations with others (pregnant women). Also central to this ethical orientation is responsiveness in an interconnected network of needs, care and prevention of harm.

This review concludes by stressing that many human relationships involve persons who are vulnerable, including pregnant women, dependent, ill and or frail, noting that the desirable moral response is that prescribed by care ethics, which thus has implications for the practice of medicine in Nigeria.

## Introduction

Poverty exists when people lack the means to satisfy their basic needs. These may be defined narrowly as "those needs necessary for survival"[1] or broadly as "those needs reflecting the prevailing standard of living in the community"[2]. Reproductive ill health is both a cause and consequence of poverty [3]. Sexual and reproductive health problems account for approximately 20 percent of the ill-health of women globally, and 14 percent of men due to lack of appropriate sexual and reproductive health services [4]. It is against this background that this review examines poverty as a major cause of maternal deaths in Nigeria. It offers an insight into the practice of medicine in Nigeria which is different from that of modern medicine because it falls short of the principle of showing care. The consequence of this is the uncaring attitude of many health care providers in the context of maternal care. It suggests ways of reducing maternal deaths in Nigeria, emphasizing at the same time an ethical orientation which is more viable and has implications for the practice of medicine in this country. This orientation – the ethics of care- emphasizes the need to show care to vulnerable patients (for example, pregnant women) irrespective of their socio-economic status. This will enhance quality care and foster good relationships between health-care providers and their patients.

### Defining the problem

Poverty can be defined as the situation of people whose "resources (material, social and cultural) are so limited as to exclude them from the minimum acceptable way of life in the countries in which they live"[5]. It is a multifaceted condition. It has many dimensions, among which are poor access to public services and infrastructure, unsanitary environmental surrounding, illiteracy and ignorance, poor health, insecurity, voicelessness and social exclusion, as well as low levels of household income and food insecurity [6]. All these aspects of poverty are life-shortening, involve great suffering and pain (from disease and hunger) and they undermine an essential dignity and decency to life [7].

It has been estimated that in 2001, 1.1 billion people had consumption levels below $1 a day and 2.7 billion people lived on less than $2 a day [8]. Poverty endangers the health and lives of many in developing countries since the most widespread and severe poverty occurs in countries such as Nigeria, Togo, Liberia and so on. At present, Nigeria's entire economy revolves around oil with large reserves. This implies that the country has, in theory, the potential to build a very prosperous economy. In fact Oil accounts for nearly 80 per cent of government revenue in Nigeria, 90–95 per cent of export revenue and over 90 per cent of foreign earnings [9]. But unfortunately, poverty is widespread in this country in spite of its rich natural resources to the extent that indicators place it among the twenty poorest countries in the world [10]. According to the World Bank, about 66 per cent of the Nigerian population now falls below the poverty line of about a dollar a day compared to 43 per cent in 1985 [11]. The wealth from oil has not feel through many sectors of the economy especially the health sector because poverty is still a growing problem in Nigeria, a country which is estimated to have earned about 280 billion U.S dollars from oil during the past thirty years [12].

Nigeria is reported to have one of the highest occurrences of maternal mortality in the world with figures ranging from 704 to 1,500 [13,14] maternal deaths per 100,000 live births. Figures based on the results of the 1999 Multiple Indicators Cluster Survey (MICS) show a wide variation from 166 per 100,000 live births in the South West to 1,549 per 100,000 live births in the North East, with a national average of 704 deaths per 100,000 live births [15].

### Causes of maternal mortality in Nigeria

More than 70 percent of maternal deaths in Nigeria are due to five major complications: hemorrhage, infection, unsafe abortion, hypertensive disease of pregnancy and obstructed labour [16]. Also, poor access to and utilization of quality reproductive health services contribute significantly to the high maternal mortality level in Nigeria. According to the 2003 Nigeria Demographic and Health Survey, 30 percent of Nigerian women cited the problem of getting money for treatment, while 24 percent cited the problems of accessibility to health facilities and transportation [17]. Also, 17 percent reported the problem of not getting a female provider in the hospital, while 14 percent reported the problem of not wanting to go alone. Again, 14 percent reported the problem of ignorance of where to go for treatment, while one in ten women complained of the bottlenecks in getting permission to visit hospitals [18].

Majority of births in Nigeria (66 percent) occur at home [19] and only one-third of live births during the five years preceding the most recent demographic health survey occurred in a health facility [20]. A smaller proportion of women receive postnatal care, which is crucial for monitoring and treating complications in the first two days after delivery [21]. Only 23 percent of women who gave birth outside a health facility received postnatal care within two days of the birth of their last child [22]. More than seven in ten women who delivered outside a health facility received no postnatal care at all [23].

The Nigerian health system as a whole has been plagued by problems of service quality, including unfriendly staff attitudes to patients, inadequate skills, decaying infrastructures, chronic shortages of essential drugs and the well-known "out-of-stock: syndrome" [24]. In some hospitals, equipment such as sphygmomanometers, thermometers, weighing scales, delivery kits, waste bins and mucus extractors are unavailable. Many do not have regular supply of electricity because they cannot maintain a standby generator. Some do not even have a regular water supply and thus require their patients to provide their own water. Coupled with all these, staff are demoralized by inadequate and irregular remuneration [25]. Many have relocated to industrialized countries where they will be adequately remunerated.

Evidence exists on the relationship between the density of health workers and maternal mortality rates in Nigeria [26]. Slightly more than one-third of births in Nigeria are attended by doctors, trained nurses and midwives [27]. This is in spite of the fact that the level of assistance a woman receives during delivery can reduce maternal deaths and related complications. The attitude of many nurses/midwives towards pregnant women and those in labour is poor. In the course of their professional duty as nurses/midwives, they act inappropriately to the woman in labour. Such attitude raises the question of what the duties of a nurse/midwife are to a woman in labour. Sometimes, one wonders if they have any knowledge of the literal meaning of their profession or even what their profession entails. The situation becomes worse if the woman in labour is an HIV-positive patient. Their attitude ranges from that of neglect to abandonment such that one questions the professional training they had and knowledge about the mode of transmission of HIV and the skill to prevent it [28].

However, the fact that health facilities physically exist in the sense of bricks and mortar do not necessarily mean that they are functional. Many hospitals in Nigeria are poorly equipped and lack essential supplies and qualified staff [29]. In fact, a 2003 study revealed that only 42 percent of public facilities in Nigeria met internationally accepted standards for obstetric care [30]. The health sector as a whole is in a dismal state. In the year 2000, the World Health Organization ranked the performance of Nigeria's healthcare system 187^th ^among 191 United Nations member states. [31]. The prevailing problems then are still persistent and are yet to be addressed due to Nigeria's long-standing socio-economic situation and crises of leadership [32].

User charges coming at a time of spreading deepening poverty have become a great barrier to access for many Nigerian women who are not educated, and hence economically disempowered. Getting money for treatment was the problem most commonly reported by Nigerian women of all backgrounds [33]. There is a strong negative correlation between both levels of education and wealth quintile. For instance, 41.6 Nigerian women had no education at all while 21.4 had primary education. 31.1 had secondary education while only 5.9 had higher education [34]. The lowest wealth quintile for women is 68.7, the second being 63.3, the middle49.2, the fourth 29.2 and the highest was 5.8 [35]. It is worthy of note to point out that even educated women may not have access to healthcare either due to the problem of poor attitude of health care providers or that of proximity to quality health care facility.

Graham, Fitzmaurice, Bell and Caims [36] examine the link between poverty and maternal mortality. According to them, there is evidence of major differentials in access to and uptake of maternity services across a wide variety of developing countries, and recently a technique has been developed to expose similar discrepancies in the risk of maternal mortality. These risks, they explain:

are a culmination of disparities in underlying health status differential lifetime exposure to pregnancy, different access to the means to avoid unwanted pregnancy, unequal physical, economic and social access to preventive services for normal pregnancy and delivery and major discrepancies in utilization of quality emergency obstetric care [37].

Poverty greatly amplifies every other risk factor for maternal mortality and morbidity from grotesque female oppression to maternal undernutrition and to inadequate medical and physical infrastructure [38]. The synergistic interrelationship between poverty and maternal mortality calls for the need to address this reproductive health problem.

### Reducing maternal mortality in Nigeria

Tracking changes in maternal mortality in developing countries such as Nigeria can be difficult, because the data are unreliable. Vital registration systems in rural areas of most developing countries are deficient and surveys produce estimates with wide margins of uncertainty [39]. Nevertheless, strategies need to be more appropriately focused this will enable pregnant women in whom complications develop have access to the medical interventions of emergency obstetrical care [40].

Programs that are likely to make such care more widely available involve upgrading rural health centers and referral hospitals and stocking them with the necessary drug supplies and equipment, such as magnesium sulphate for eclampsia, antibiotics for infection and basic surgical equipment for cesarean sections. Coupled with this is the need to train cadres of health workers and develop strong referral systems between communities and health care facilities, since delays in care can be life threatening [41]. A referral system includes means of communication and transport as well as mechanisms for ensuring that referral facilities are able to provide services at all hours.

However, when a functioning health-care system is in place, some interventions at the community level, such as the use of misoprostol to strengthen contractions, help expel the placenta and control bleeding before transfer to a health care facility could contribute to significant reduction in maternal mortality. The effective functioning of any facility will depend on whether pregnant women have skilled attendants at delivery–an accredited health care professional (e.g. a doctor, midwife or nurse) who can conduct normal deliveries, identify and manage complications, and refer women to the next level of care [42].

There is need for skilled attendants to be well remunerated in order to improve performance and efficiency. Ministries of health in Nigeria also have roles to play in strengthening their health care systems and address inequality because resources often fall short of the minimum levels needed. Ensuring equitable distribution of the new found wealth accrued from oil is a viable recommendation here. The government's main challenge should include improving the health sector especially maternal health without which many pregnant women in Nigeria will continue to face the problem of access to health care which may increase the prevalence of maternal mortality.

Lastly, there is need to empower many Nigerian women. Pregnant women in low resource countries such as Nigeria, Togo, Liberia and so on often incur catastrophic costs to obtain the care they need [43].

The governments of developing countries, notably Nigeria, must establish supportive policies. Although every country has its own history and challenges, accelerating progress is not impossible if political will can be translated into action. How then can this be achieved? Maternal mortality has been described as the symptom of an underlying disease: the country's shambolic socio-economic and political systems, which result in very poor obstetric care [44]. This disease must be treated. Devoting resources to a single symptom such as maternal mortality will at best lead to temporary amelioration. There is need for health-care providers to imbibe a more viable ethics of the medical profession.

### Care ethics as a health care practice in Nigeria

The writings of Carol Gilligan [45] and Nel Noddings [46] comprise two widely recognized care-focused feminist approaches to ethics. In her pathbreaking book, *In a Different Voice *[47], Gilligan emphasizes the unique form of moral reasoning that caring engenders. Gilligan's target, Tauber explains, is the Kantian tradition that espouses justice as the first moral principle and the psychological schools of Piaget and Kohlberg whose studies support the moral ideas of individual rational will or autonomy [48]. In Gilligan's empirical studies, she discovers that the standard of what is moral determines moral development, and if these standards follow a particular masculine trajectory, women fare less well when assessed [49]. She argues that perhaps another standard of ethics might be applied; one that stresses different values – connections, relationships and reciprocity and within such a moral system women prevails over men. Thus, the ethics of caring is contrasted with the ethics of justice.

By contrasting this feminist moral compass with male ethos stressing independence and separateness, Gilligan presents a strong challenge to older versions of individualism. The response to dependency and to the need for interconnections between people portrays a social universe characterized by interrelated actor not atomic ones. Thus, the ethics of caring was born as a product of an understanding about persons in a social web designed to create harmonious interactions [50]. Since it shares some features with the traditional African communal value of everyone being his/her brother's keeper, such positive societal value need to be re-invented in the context of the patient-physician relationship. Traditional African societies are famously communalistic. The individual is brought up from the beginning with a sense of belonging and solidarity. The basis of this solidarity is a system of reciprocity in which each individual has obligations to a larger set of other individuals. Extending this traditional outlook to the physician-patient interaction will be a valuable contribution to the practice of medicine in Nigeria.

In a similar vein, Nel Noddings in her *A Feminine Approach to Ethics and Moral Education *[51] notes that ethics is about personal relations defined as "a set of ordered pairs generated by some rules that describe the effect or subjective experience of the members" [52]. She continued by explaining that:

There are two parties in any relation. The first member is the one caring; the second, the "cared-for" if the relation between these two parties is a good one, the one-caring is motivationally engrossed or displaced in the cared for. She or he attends to the cared-for not only in thoughts, however but also in deeds. When a caring relationship succeeds, the cared-for actively receives the caring thoughts and deeds of the one-caring, spontaneously sharing her or his aspirations appraisals, and accomplishments with her or him. [53].

For Noddings, caring is not about feeling favourably disposed towards human beings in general; rather it is about concrete interactions between particular persons. Her argument starts from the position that care is basic in human life- that people want to be cared for. According to her,

Ethical caring, the relation in which we do meet the other morally arises out of natural caring- that relation in which we respond as one-caring out of love or natural inclination. The relation of natural caring is the human condition that we consciously or unconsciously perceive as "good". It is that condition toward which we long and strive, and it is our longing for caring–to be in that special relationship–that provides the motivation for us to be moral. We want to be moral in order to remain in the caring relation and to enhance the ideal of ourselves as one-caring [54]

Her approach is to examine how caring is actually experienced (what we might describe as a phenomenological analysis). She asks: "what are we like when we engage in caring encounters?" Perhaps the first thing we discover about ourselves, she continues, is that we are receptive; we are attentive in a special way. Receptive attention is an essential characteristic of a caring encounter. The care-giver is open to what the cared-for is saying and might be experiencing and is able to reflect upon it [55].

However, motivational displacement, the care-giver's 'motive energy', flows toward the 'cared-for'. The care-giver thus responds to the cared-for in ways that are hopefully helpful. For this to be called caring, a further step is required: some recognition on the part of the cared-for that an act of caring has occurred. Caring involves connection between the care-giver and the cared-for and a degree of reciprocity. This implies a mutual relationship between the care-giver and the cared-for in which the cared-for is expected to respond in the appropriate way to the care-giver's caring attitude. Noddings argues that caring-about needs more attention. According to her:

We do not have to construct elaborate rationales to explain why human beings ought to treat one another as positively as our situation permits. Ethical life is not separate from and alien to the physical world. Because we human beings are in the world not mere spectators watching from outside it, our social instincts and the reflective elaboration of them are also in the world [56]

Care ethics rests on the understanding of relationships as a response to others in their terms. This ethical orientation can be a morality with universal appeal not only because it emphasizes the need to cultivate our natural capacity to care for others and ourselves but also because it stresses values such as care, trust, mutual consideration, sympathy and the like. These also extend to activities such as childcare, antenatal care, health care, childbirth and, medical practice generally. It shows the potentials of this ethical orientation for dealing with global problems in health care.

Caring is an ethical obligation inherent in the health providers' role. However, caring transcends role obligations. It acknowledges the vulnerable humanness of the other and reinforces the caring of the career [57]. Ethical principles such as ***Beneficence, non-maleficence, autonomy and justice ***are not at variance with care: they provide specific judgments in the context of caring for another person [58].

***Beneficence (Do Good)***, which primarily emphasizes enhancing kindness, charity and the welfare of others, is intervened with ***non-maleficence (Do No Harm)***. Non-maleficence has been described as the primary admonition of the Hippocratic Oath:

A physician ought not to inflict pain, suffering and distress (physical or psychological), loss of freedom, disability and death. An individual should not deprive others from pleasures and happiness by restricting autonomy" [59].

Bill Fullford, Dickenson and Thomas [60] describe these principles as *Prima Facie; *that is they are principles that are likely to be relevant in some degree to any given ethical problem in practice. However, "Do No Harm", the primary admonition of the Hippocratic Oath, is the ethical code of the medical profession in Nigeria. The ethic of competent care can also be called Hippocratic ethic, after Hippocrates (c. 460-378 B.C.E), the "father of medicine". One phrase in the Hippocratic oath " I will act for the benefit of my patient according to my ability and judgment" implies the imperative of the competent practice of the art of medicine. [61].

Unfortunately, the Nigerian health-care providers have failed in their duty to use their knowledge for the reduction of suffering for patients particularly pregnant women. Due to the poor remuneration doctors receive, their attitude to work has been negatively affected. Many of them float their private hospitals where they spend most of their time. The consequence of this is the inavailability of health-care providers in state and federal hospitals. In most cases, patients find themselves apologizing for the inconvenience of needing attention because, according to them, the doctor knows best.

Since many skilled and competent health care providers have relocated to industrialized countries due to poor pay and poorly equipped hospitals, (which does not give room for efficient performance) some of the few doctors on ground are in the habit of referring many pregnant women to their private hospitals in order to make more money. Private hospitals are expensive in Nigeria, and pregnant women who cannot afford them resort to mission houses [62]. This puts such women at risk of maternal mortality [63].

The Nigerian health sector has often allowed a rule of ability to pay to determine the distribution of health, goods and services. But as Beauchamp and Childress argue, rules like this should not serve as the only principle of distributive justice.

According to them,

we do not always need to place a monetary value on human life. In many cases, qualitative factors are more important than purely economic factors. For example how some deaths occur, by what means they occur and with what symbolic features may legitimately lead a society to allocate its resources differently in order to reduce various risks of death [64].

However, a 27-year-old woman from the northern part of Nigeria who came on a visit to a family friend and could not afford the initial payment of an emergency bill complained of neglect, and consequently delivering her baby in a vehicle. These problems may stem from a failure to teach and nurture empathy in medical education and from financial incentives that discourage spending time at patients' bedsides and getting to know patients as persons.

Care ethics can impact on the outcome of medical practice in Nigeria in many ways. First, the ethics of care may lead to positive change in biomedical education including placing greater emphasis on health-care providers, communication skills and emotional sensitivity and on the effects that ethical issues have on relationships [65].

In addition to producing changes in ethics education, a care orientation arguably requires placing greater emphasis on beneficence as the healthcare providers' primary responsibilities to the patient. Since caring for patients represents a central component of ethics in medicine, caring is inextricably linked to the physician's obligations to relieve suffering. There is, therefore the need to educate Nigerian doctors on the imperative of care ethics.

Again, care ethics becomes manifest in the practice of Medicine in the activity of healing the patient. The practice of medicine in Nigeria construes the injunction "respond to the need and vulnerability" hearing it as a call to find the proper cure for diseases. Care ethics understands this injunction as a broader moral requirement that health care providers should address both the physiological and psychosocial needs and vulnerabilities brought about by illness or pregnancy. Here the physician incurs a duty of beneficence; a duty requiring the physician to respond to the patient's needs and promote the patient's good. Other ethical values can be derived from the physician's primary duty of beneficence [66].

Additionally, the focus on care and relationship takes on particular importance with a particular trend, promising to radically transform the face of medical practice particularly in Nigeria. By bringing into focus important issues relating to the increasing number of pregnant women among the patient population in Nigeria, care ethics demonstrates that its contributions to the field of medicine in this country have practical and not just theoretical importance. In the care of pregnant women, for example, the concern raised by care ethics takes on added importance. By engaging in acts of care such as being present to the pregnant woman, both before and during delivery, listening to her fears and concerns, providing support and encouragement and tending to her physical comfort, the needs of the pregnant woman are addressed. At the same time, health care providers in Nigeria are provided with an avenue for attending to the needs of their patients. This would help alleviate the pregnant women's feelings of anxiety about the reality of the child, his/her well-being and other experiential variables that affect women's reactions in pregnancy. These include the uncertainty about when labour will begin, what labour pain is like, (especially for the inexperienced mother-to-be), and the outset of labour coupled with the obstetric management of the birth itself.

Thus, the discussion on Care ethics suggests that the concerns raised by this ethical orientation in the medical context will become an even more important aspect of medical practice in Nigeria as the percentage of pregnant women in the patient population increases. This area of overlap between care ethics and medical practice suggests that care ethics has implications for medical practice in Nigeria.

Despite the integral role that an ethic of care plays in medicine, this ethical orientation is beset with some limitations. Identifying some of these limitations, Sokol D.K. explains that certain factors place limits on the health worker's duty to care. They include the working environment, the health-care worker's specialty (he/she may not be a specialist in the field of obstetrics and gynecology) which may also affect knowledge of the likely harm and benefits of treatment and the conflicting obligations arising from the health worker's multiple roles [67]. Also, care ethics threatens to undermine the hard-won concern with patient autonomy because it rests on the assumption that the care-giver, simply by virtue of caring, knows what is in the best interest of the care recipient.

Lastly, an ethic that requires the active cultivation of caring relations with patients may seem unworkable given the realities of large urban hospitals and managed care, both of which limit the amount of time health care practitioners may spend with their patients. Moreover, even if there is the time necessary to cultivate such caring relations, the demands of care ethics might seem too taxing and too likely to result in burn-out on the part of caregivers to be widely advocated [68].

The public and patients alike should cultivate tolerance as a character trait. This virtue allows patients to acknowledge the physician's multiple roles. Some forms of dialogue between the public and the medical profession, through the media, public consultations and educational establishments could help establish a mutually acceptable set of limits.

In spite of these limitations, care ethics can enhance the health of the population in several ways especially when one considers its major feature which involves having a certain emotional attitude and expressing the appropriate emotion in action. This is a major requirement of the care ethics perspective which views partiality toward others as not only morally permissible but an expected norm of interaction. Describing it as an ineliminable feature of the human condition, Beauchamp and Childress explain that without exhibiting partiality, we stand to sever important relationships and to alienate others [69]. The care perspective is especially meaningful for roles such as parent, friend, physician and nurse in which contextual response, attentiveness to subtle clues, and deepening special relationships are likely to be more important morally than impartial treatment [70]

Also, if subsumed under virtue ethics, care ethics can improve and enhance the health of the population. Raja, H. explains that by construing care ethics as an important virtue, the features of care ethics such as particularity, partiality, emotional engagement and the importance of care to our moral lives will be preserved [71]. Since virtue ethics emphasizes developing good character traits, developing the above mentioned qualities would involve practicing them. A physician cannot become virtuous if he routinely fails to fulfill his moral obligations or duties to care.

Again, care ethics can improve the health of the population if patients and physicians alike have an accurate notion of not only what it means to be human but also what it means to be a healthy human. Health needs must not be subordinated to human needs. Without a sufficiency of health, we cannot satisfy any of the other needs such as material goods and security. Ethics concerns the needs and values of human persons. Health is a vital human need; nothing is more human, more personal and must always be one of the main concerns of any human community [72].

Although physicians belong to their own professional community and adherence to its set of rules, they are also part of the broader community and are therefore subjected to the same rights and duties as other members. The two spheres of obligations- professional and personal- are both separate and overlapping [73]. They are separate because their obligations as physicians toward their patients give them rights that non-medical members of the society do not possess. The spheres are overlapping, however, in that their role as doctors does not completely absolve their responsibilities as members of a broader community [74].

A collective 'we-self' sense of personhood may provide a more practical and helpful ethical approach, one which strives to balance the needs of the individual with those of the larger independent whole [75]. In Nigeria's current scarce resources scenario, we may find wisdom in the way traditional "we-self" culture has developed ethical guidelines for solving various problems.

Finally, patients also have a duty to care for healthcare workers. Part of this duty is not to require doctors to transcend the bounds of reasonable risks during treatment and to respect and acknowledge their roles outside the realm of medicine.

## Conclusion

This paper has been able to examine how poverty impairs women's health and hinders human development. It highlights the internal and external factors responsible for maternal deaths in Nigeria and also suggests practical ways of reducing maternal mortality in this country. The paper drew on the relevance of care ethics as an ethical orientation that can resolve logistic problems such as delay in the Nigerian health care system, which often results in poor reproductive health care services. It concluded by stressing that partiality which is a practical aspect of an ethical orientation known as care ethics, has implications for medical practice in Nigeria. This is because its commitments to values such as showing care, responsiveness and concern for the needs of the vulnerable (such as pregnant women) are consonant with the practice of medicine.

## Competing interests

The author declares that she has no competing interests.
